# 174. Implementation of Methicillin Resistant Staphylococcus Aureus (MRSA) Nasal Polymerase Chain Reaction (PCR) Swabs in Pediatric Patients

**DOI:** 10.1093/ofid/ofae631.054

**Published:** 2025-01-29

**Authors:** Hannah Bischoff, Heather Hughes, Beth Addington, Sarah Withers, Katherine Richardson, Carmen Faulkner-Fennell

**Affiliations:** Prisma Health Upstate - Greenville Memorial Hospital, Greenville, SC; Prisma Health Upstate - Greenville Memorial Hospital, Greenville, SC; Prisma Health Upstate - Greenville Memorial Hospital, Greenville, SC; Prisma Health, Greenville, South Carolina; Prisma Health Upstate - Greenville Memorial Hospital, Greenville, SC; Prisma Health Upstate - Greenville Memorial Hospital, Greenville, SC

## Abstract

**Background:**

Current society guidelines for community-, hospital-, and ventilator-acquired pneumonia in adult patients mention use of polymerase chain reaction (PCR) nasal screening for methicillin-resistant *Staphylococcus aureus* (MRSA) colonization to aid de-escalation of anti-MRSA antibiotics. MRSA nasal PCR has a 96% negative predictive value when utilized for pneumonia. Most available data is in the adult population with limited application in the pediatric patient population. In a single-center retrospective analysis of 95 pediatric patients, MRSA nasal PCR showed a negative predictive value of 95.5% in multiple types of infections. The objective of this study is to expand on the limited pediatric data, encouraging the use of MRSA nasal PCR as a tool to guide de-escalation of anti-MRSA antibiotics.

**Methods:**

This single-center, pre- and post-interventional, retrospective cohort study compared antibiotic regimens in pediatric patients who were treated empirically with anti-MRSA antibiotics and had a MRSA nasal PCR collected compared to those who did not have a MRSA nasal PCR collected. Use of MRSA nasal PCR in the pediatric hospital was encouraged following an antimicrobial stewardship provider-lead continuing education presentation. The primary outcome was duration of therapy of anti-MRSA antibiotics in days. Secondary outcomes include positive predictive values (PPV) and negative predictive values (NPV) for all infections, pneumonia, and skin and soft tissue infections.

**Results:**

A total of 319 patients were included in the study, 252 in the pre-intervention group and 67 in the post-intervention group. The duration of anti-MRSA antibiotics in the pre-intervention group was 6.66 days compared to the post-intervention group at 2.00 days (p-value = 0.027). Using data from 38 patients with appropriate culture results for the diagnosis, negative predictive value was calculated as 92.1%. Skin and soft tissue infections and pneumonia were found to have negative predictive values of 90.1% (22 patients) and 100% (5 patients), respectively.

**Conclusion:**

Implementation of MRSA nasal PCRs swab in pediatric patients significantly reduced the number of days patients received anti-MRSA coverage promoting their use for antimicrobial stewardship.

**Disclosures:**

**All Authors**: No reported disclosures

